# Design, implementation, and evaluation of an online supported peer feedback module to enhance students’ argumentative essay quality

**DOI:** 10.1007/s10639-023-11683-y

**Published:** 2023-03-15

**Authors:** Omid Noroozi, Seyyed Kazem Banihashem, Harm J. A. Biemans, Mattijs Smits, Mariëtte T.W. Vervoort, Caro-Lynn Verbaan

**Affiliations:** 1 Wageningen University and Research, Wageningen, The Netherlands; 2 The Open University, Heerlen, The Netherlands

**Keywords:** Argumentation, Essay writing, Higher education, Online learning, Peer assessment, Peer feedback

## Abstract

We know little to what extent peer feedback strategies can be applied on a large scale in higher education for complex tasks. This study aimed to design, implement, and evaluate an online-supported peer feedback module for large-scale use to enhance higher education students’ argumentative essay writing performance. To do this, 330 students from five different courses at bachelor and master levels followed the online supported peer feedback module. In this module, students were asked to write an argumentative essay about a controversial issue, provide peer feedback for two peers, and revise their original essays based on the received feedback. Three types of data including original essay (pre-test) data, peer feedback data, and revised essay (post-test) data collected. Students also filled out the learning satisfaction questionnaire at the end of the module. The findings showed that the suggested online-supported peer feedback module was effective in improving students’ argumentative essay quality in all courses at the bachelor and master levels. The findings also showed there is a difference in the level of students’ satisfaction with the module among the courses and between the education levels. The findings of this study provide insights into and add value to the scalability of online peer feedback tools for argumentative essay writing in different contexts. Based on the findings, recommendations for future studies and educational practice are provided.

## Introduction

Argumentation is a critical skill for scientific practice in higher education (Fan & Chen, 2021). Within higher education contexts, educators expect students to be able to critically think about a controversial issue, involve in an argumentation, claim a position, defend their positions with scientific arguments, facts, and evidence, and respond to the counter-arguments (Lazarou et al., 2016; Toulmin, 1958). Previous studies indicate that argumentation contributes to students’ development of thinking skills and domain knowledge (Mayweg-Paus et al., 2021; Ogan-Bekiroglu & Eskin, 2012;), learning achievements (Akbas et al., 2019), and academic performance (Foutz, 2018). Usually, students in higher education practice argumentation skills via wiring essays (Liunokas, 2020). However, the scientific evidence shows that writing a good argumentative essay is not an easy task for most higher education students and they usually fail to perform at a satisfactory level due to different reasons such as difficulties to understand the structure of a high-quality argumentative essay (e.g. Butler, 2011), lack of domain-knowledge (e.g., Alqassab et al., 2018; Valero Haro et al., 2019), or challenges in transforming the argumentation knowledge into the application (e.g., Valero Haro et al., 2022).

Peer feedback is a promising learning strategy that has been used for improving students’ argumentative essay writing in higher education (Awada & Diab, 2021; Baker, 2016; Jongsma et al., 2022; Latifi et al., 2021). Peer feedback is important particularly within online settings, especially where the class size is much bigger and it is challenging for teachers to give effective one-by-one feedback due to immersive high workload and shortage of time (Er et al., 2021). In prior studies, peer feedback was typically provided in a supported and structured way as students struggle with delivering high-quality feedback all on their own (Noroozi et al., 2016; Kerman et al., 2022) because most of the students are not well aware of the features and structure of good peer feedback and how to provide quality peer feedback on their peers’ work (Er et al., 2021; Kerman et al., 2022; Nelson & Schunn, 2009; Ramon-Casas et al., 2019). This raises a need to provide support and guidance for higher education students in giving feedback on their peers’ work.

A review of the literature shows that using supported peer feedback in online settings to improve students’ argumentative essay writing was effective (e.g., Latifi et al., 2021), however, its impacts were influenced by other variables, more specifically, students’ domain knowledge and their prior experiences that can differ between bachelor and master students (e.g., Alqassab et al., 2018; Vale Haro et al., 2019, 2022; van Zundert et al., 2012). This means that students’ peer feedback performance on essay writing could differ depending on their level of domain knowledge and whether they are experienced in providing peer feedback or not. This creates a problem in terms of the scalability of the supported peer feedback tools in higher education. The available online supported peer feedback tools tend to be context-specific which means they can not be used in different courses for different education levels since students’ domain knowledge and experience vary from one to another context. Therefore, teachers have to either design and develop their own specific peer feedback tool or adjust the available peer feedback tools to use in their courses. For teachers, this means more workload and spending more time and effort on designing peer feedback. Since, teachers already face a huge workload in higher education (Shi, 2019), this can result in giving up on the effective use of peer feedback tools. This important issue in the literature raises an urgent need to explore how the use of supported peer feedback tools can be scaled up in online settings. To address this research gap in the scientific literature, in this study, we aimed to design and develop an online supported peer feedback tool that can be used on a large scale for different course domains and different education levels. In addition, we aimed to investigate students’ satisfaction with the designed peer feedback tool and their experiences during the learning process because satisfaction is a key variable in the effective implementation of any learning activity including peer feedback activity and if students do not feel satisfied with the adopted peer feedback activity, they will not successfully uptake it (Mercader et al., 2020).

### Online peer feedback

Peer feedback is defined as a process where students generate oral or written feedback on their peers’ work and also receive feedback from peers on their own work (Topping, 1998). The purpose of providing feedback is to help peers to know what are the issues in their work and how they can fill the gap between the current level of performance and the desired level (Boud & Dawson, 2021; Foo, 2021). Studies have shown that peer feedback could have a very strong impact on learning (Banihashem et al., 2022; Liu & Carless, 2006; Topping et al., 2000) if it is delivered timely and with high quality (Banihashem et al., 2021; Patchan et al., 2016). High-quality feedback entails features such as words of compliment for good work which is usually called affective feedback (Foo, 2021; Wu & Schunn, 2020). In addition, good feedback should include cognitive and constructive comments which refer to identifying issues and gaps in the work alongside suggestions for improvements of the work (Patchan et al., 2016; Wu & Schunn, 2020).

In recent years, the interest in using peer feedback to reflect on students’ work and facilitate learning in online environments within higher education contexts has been growing significantly (Huisman et al., 2019; Iglesias Pérez et al., 2022; Noroozi et al., 2016, 2022; Wood, 2022). One main reason relates to the growing number of students in higher education settings who follow online courses (Er et al., 2021). It is reported that class sizes continue to grow every year in higher education contexts (Shi, 2019). For such large-size online classes, effective feedback from educators on every single work of individual students requires an extreme workload (Nicol & Macfarlane-Dick, 2006). While, peer feedback as a scalable and effective learning strategy can be used for improving students’ quality of work in online classes with a large cohort of students (Er et al., 2021; Kerman et al., 2022). In online learning environments, students can provide feedback synchronously and asynchronously on their peers’ work in both oral and written formats (Shang, 2017). However, asynchronous and written peer feedback is the most widely used form of online peer feedback (Foo, 2021). One reason why written and asynchronous feedback is the most popular form of feedback is because of the complexity of providing effective and constructive feedback which causes cognitive workload and requires students to take their time and carefully think about it (Valero Haro et al., 2019). Asynchronous communication provides time to reflect and better analyze information (Veerman et al., 2000). Peer feedback helps students to be actively involved in online learning activities and without peer feedback students are more likely to be disconnected from online classes compared to F2F classes (Ko & Rossen, 2017). The scientific evidence shows that the implementation of peer feedback in online classes is positively associated with the quality of dialogue and discourse (Ertmer et al., 2007), learning (Liu & Carless, 2006), and community building (Corgan et al., 2004). In recent years, one of the complex learning tasks in higher education that online peer feedback is being increasingly used for is argumentative essay writing (e.g., Huisman et al., 2019; Jin et al., 2022; Latifi et al., 2021).

### Online peer feedback for argumentative essay writing

Higher education students typically practice their argumentation skills by writing an argumentative essay (Liunokas, 2020). Argumentative essay writing is a critical learning task for students as they can practice how to provide claims on a controversial issue that could be scientifically convincing, how to support claims with evidence and facts, and how to provide valid responses to possible counter-arguments (Lazarou et al., 2016). According to the literature, a high-quality argumentative essay should begin with an introduction on the topic, claiming a position, presenting argumentation in favor of and against the position, responding to the counter-arguments, and finally making a conclusion (Toulmin, 1958; Wingate, 2012). Composing such a high-quality structure in argumentative essay wiring is challenging for students and previous studies suggest that students need support on how to write a good argumentative essay (e.g., Noroozi et al., 2016; Latifi et al., 2020). Online peer feedback has the potential to enhance students’ argumentative essay writing performance (Huisman et al., 2019; Zhang & Zou, 2022). Through peer feedback activity, students focus on reading their peers’ essays, reviewing the quality of peers’ essays, identifying problems, and suggesting points for improvements (Lizzio & Wilson, 2008; Topping, 2009). Students who received peer feedback can implement the comments in their essays to improve the quality of their essays. However, providing effective online peer feedback requires higher-order thinking skills (Kern et al., 2003; King, 2002). For high-quality feedback, students need to have critical thinking skills to identify problems (Xiong & Schunn, 2021) and high cognitive thinking skills to provide constructive comments on how to address the problems in their peers’ work (Noroozi et al., 2022). Studies have shown that students struggle with delivering effective peer feedback and usually, it remains at the surface level (Er et al., 2021; Ramon-Casas et al., 2019; Wu & Schunn, 2020). Students’ feedback is typically not well-founded with solid arguments and it mostly focuses on personal qualities rather than instructional qualities and learning goals (Hattie & Timperley, 2007). This means that most of the students are not fully aware of how and on what feedback should be given in essay writing tasks. Scholars suggest supporting students in online peer feedback activities in order to prevent superficial peer feedback performance (e.g., Gielen & De Wever, 2015; King, 2002; Zhao, 2014).

### Supported online peer feedback for argumentative essay writing

The quality of feedback provided by students is uneven (Nilson, 2003) as students’ expertise in and experiences with providing feedback are varied (Gielen et al., 2010). Supported peer feedback with clear criteria can help all students to become aware of the structure of good feedback and how to provide quality feedback on their peers’ essays (Gielen & De Wever, 2015; Ramon-Casas et al., 2019; Tsai & Chuang, 2013). When students are guided with a clear structure for feedback, they focus more on the content, structure, and quality of the argumentation in essay writing instead of peers’ personal characters (Gielen et al., 2010). Although prior studies highlight that supported peer feedback improves students’ performance in argumentative essay writing (Latifi et al., 2021; Noroozi et al., 2012; Ramon-Casas et al., 2019), the missing point in the literature is that most peer feedback studies focused on a single variable and took a variable-oriented approach which does not fill the scientific research gap in providing a comprehensive understanding of how a peer feedback tool can be used on a large scale in different courses at different educational levels and in different content domains. In other words, the suggested peer feedback tools in the literature were context- and content-oriented as they were implemented in one course domain or one education level and this weakens these tools’ capacity to be scaled up for other contexts. (Ramon-Casas et al., 2019; Schillings et al., 2021; Zhao, 2018). For example, only 52 undergraduate students from one course participated to test a scripted peer feedback tool (Latifi et al., 2021). Similarly, Zhao’s (2018) study only covered 18 undergraduate students, and Schillings et al.’s (2021) study was conducted with 84 students from one course. This indicates that we lack knowledge and evidence on the effectiveness of the peer feedback tools that could be applied on a large scale for students with different domain knowledge at different education levels. This is striking especially when we notice that in higher education, the class sizes continue to grow every year (Shi, 2019) and it becomes more difficult for teachers to provide one-by-one feedback (Noroozi & Hatami, 2022). Due to this high workload and lack of time, teachers are in grave need of peer feedback tools that can be used without a lot of adjustments. In the context of argumentative essay writing, this is more important since teachers have to spend more time and effort giving feedback due to the complex nature of argumentation, and using a peer feedback strategy is urgent in such context (Latifi et al., 2021).

Maybe one would argue that having a peer feedback tool that can be used in different course domains and at different educational levels for improving students’ argumentative essays is not applicable since students’ argumentation performance is influenced by their domain knowledge (Patchan & Schunn, 2015; Van Zundert et al., 2012). It is true that some previous studies highlighted domain knowledge as an essential prerequisite for delivering effective peer feedback, particularly if the learning task is complex such as argumentative essay writing (e.g. Alqassab et al., 2018; Van Zundert et al., 2012). If students do not have sufficient domain knowledge, they are expected to provide low-quality feedback (Alqassab et al., 2018). For example, a study conducted by Patchan and Schunn (2015) showed that students with low-level domain knowledge usually tend to give a compliment or praise in their feedback, whereas students with high-level domain knowledge generally provide more criticized and rich feedback. Each discipline has its own specific features, values, epistemologies, and terminologies (Andrews, 2010; Noroozi et al., 2016). However, there is evidence that supports the potential to transfer argumentation structure across different disciplines (Alqassab et al., 2018; Noroozi et al., 2018). That means that regardless of the content and nature of the course/discipline and students’ domain knowledge, the argumentation structure is comparable and it can be independently improved.

Similar to domain knowledge, another argument here could be related to education level. One could argue that master and bachelor students’ argumentation performance in essay writing can differ. Master students tend to have more domain knowledge and learning experiences in terms of academic and scientific writing on controversial topics (Van Seters et al., 2012; Yu et al., 2019) In addition, master students are more independent and critical in their works and they are expected to perform peer feedback activities with higher quality compared to bachelor students (Aghaee & Keller, 2016). However, as we explained above, not only argumentation structure can be transferred from one course/curriculum to another one but also it can be applied to different education levels.

When students are asked to write an argumentative essay, they are expected to write an introduction, take a position, present arguments and counter-arguments, respond to the counter-arguments, and make a conclusion (Chuang & Yan, 2022; Toulmin, 1958). This structure for argumentative essay writing is general and it can be followed by students with different backgrounds (e.g., study programs, and education levels) (Noroozi et al., 2018). This suggests that it is possible to provide peer feedback in different course domains if the supported peer feedback tool focuses on the structure of the argumentative essay rather than solely the content.

The review of the literature shows that implementing a supported peer feedback tool focused on the structure of argumentative essays has not been tested on a large scale and in different settings (i.e. course domains and education levels) within a higher education context in an online learning environment. Thus, there is a need to study the scalability of the supported peer feedback tools for argumentative essay writing in different content domains and education levels, so that teachers can use the tool in different course domains and education levels for improving students’ argumentation performance in essay writing. The current study can be found even more timely and important, considering its implementation within online learning environments where we have been witnessing a sharp transition to online education, especially after the outbreak of Covid-19. It has now become even more urgent to find effective online tools to tackle current challenges in student engagement and delayed feedback (Salakhova et al., 2020). Thus, this study aims to design, implement, and evaluate an online supported peer feedback module that can be used on a large scale for students from different course domains at bachelor and master levels considering their learning satisfaction.

### Research questions

The following research questions are formulated to guide this study.

- RQ1. To what extent does the supported online peer feedback module affect students’ argumentative essay writing performance?
- RQ2. To what extent does the supported online peer feedback module affect students’ augmentative essay writing performance depending on their course domains?
- RQ3. To what extent does the supported online peer feedback module affect students’ augmentative essay writing performance depending on their education level (bachelor vs. master)?
- RQ4. How do students in different course domains and education levels (bachelor vs. master) respond to the supported online peer feedback module in terms of their satisfaction?

## Methods

### Study design

This was a mixed study where different methods were employed at different phases. The designing phase had a qualitative nature where researchers collected qualitative data from the literature and experts through several meetings to design the module. The implementation phase had a quantitative nature where researchers carried out a quasi-experimental method with pre-test and post-test design to test the designed module. In this phase, students’ first essays were considered as the pre-test and the revised essays were considered as the post-test. The evaluation phase had both qualitative and quantitative nature as we used a coding scheme to analyze argumentative essay data which had a qualitative nature and we used quantitative analysis to explore the impacts of the implemented module on different selected variables for this study.

### Participants

This study was conducted during the academic year 2020–2021 in different periods at Wageningen University and Research, the Netherlands. For the sake of generalizability, we purposefully selected courses at bachelor and master levels from different course domains in Beta, Gamma, and, Beta-Gamma domains. Being able to write argumentative essays is an important and integral learning outcome for these courses. Such a large sample with diverse domains and study programs could enable us to understand the extent to which we can expand the outcomes of this study to other courses in higher education institutes that deal with controversial issues and complex problems. In total, 330 students from five different courses from different domains including Course A (Social Sciences), Course B (Plant Sciences), Course C (Health & Social Sciences), Course D (Environmental Sciences), and Course E (Food Sciences) at bachelor and master level participated of which 284 students have completed the module (Table 1). The selected courses had different natures. While four courses (courses B, C, D, and E) were compulsory and core courses related to students’ professional backgrounds, one course was an elective course that all students from different study programs could choose from several optional subjects (course A). Such diversity of the courses could also reveal how students perform with respect to domain-specific and domain-general knowledge. Students were chosen from these specific courses at bachelor and master levels for two reasons. First, each course represented a specific domain. For example, course A was from Social Sciences, while course D was from Environmental Sciences. Such variation could better help us with testing the scalability of the suggested peer feedback tool across different domains. Second, in this course, writing an argumentative essay was one of the key tasks that students were supposed to complete. Therefore, the justification for the use of the peer feedback tool in these courses was well-interpreted. To comply with the ethical considerations, this study was conducted under the supervision of the Social Sciences Ethics Committee at Wageningen University and Research. Participants were informed about the research setup of the courses, and their data has been collected with their consent. Analysis and report of the data were done anonymously.

**Table 1:** Participants’ demographic information

Course name	Degree				Gender			
	Bachelor		Master		Female		Male	
	N	Pct.	N	Pct.	N	Pct.	N	Pct.
Course A (Social Sciences)	-	-	56	100	9	39.1	14	60.9
Course B (Plant Sciences)	-	-	29	100	20	69	9	31
Course C (Health Sciences)	47	100	-	-	41	87.23	6	12.76
Course D (Environmental Sciences)	101	100	-	-	70	69.3	31	30.69
Course E (Food Sciences)	-	-	51	100	37	72.5	14	27.5
Total	148	52.11	136	47.88	195	68.66	89	31.33

### Module design and implementation

The design of the module started with a literature review of relevant works on peer feedback and argumentative essay writing to see how peer feedback was designed to improve students’ argumentative essay writing in previous studies and what is needed to design an effective peer feedback tool that aligns well with improving students’ argumentative essay writing on a large scale. Based on the literature review, we built our peer feedback tool on the work of Noroozi et al. (2016). In the previous work, Noroozi et al. (2016) offered a set of question prompts covering eight elements in line with the elements of high-quality argumentative essay writing (Nussbaum & Edwards, 2011; Toulmin, 1958). In line with this setup, we built our designed peer feedback tool on eight elements including an introduction on the topic, taking a position, arguments for the position, justifications for arguments for the position, arguments against the position, justifications for arguments against the position, response to counter-arguments, and conclusion.

Although the designed peer feedback tool was built on Noroozi et al. (2016) work, it differs from the previous work in three main ways. First, in our peer feedback tool, we started with an “introduction on the topic” which focuses on the explanation of the motivation, importance, and social aspects of the controversial topic, while Noroozi et al.’ (2016) tool starts with “intuitive opinion on the topic” which refers to a more instinctive understanding of the topic based on automatic cognitive processes. This different definition of the “introduction section” of the argumentative essay leads to a different formulation of the introduction in argumentative essay writing. While, focusing on the motivational, essential, and social aspects of the topic in the introduction section can result in framing a more inclusive introduction, drawing an introduction based on intuition may cause a threat of a very narrowed perspective in outlining the introduction section. Second, our peer feedback tool includes “taking a position on the topic”, while in Noroozi et al. (2016) tool, this element is not included. Taking a position on the topic, either in favor or against the topic, can make the argumentation claim much clearer in argumentative essay writing and for the readers. This addition to the designed peer feedback tool can make it easier for students to provide more specific feedback on the important parts of a high-quality argumentative essay. Third, in our adjusted peer feedback tool, we added “response to counter-arguments” which is a new element compared to Noroozi et al. (2016) tool. In high-quality argumentative essay writing, it is expected from students to respond to possible counter-arguments and strengthen their arguments by providing rebuttals for the counter-arguments. This can significantly improve the level of persuasiveness of the taken claim in the essay. Adding taking a position on the topic and responding to counter-arguments was based on both research and practice reasons. From the scientific point of view, it is in line with the structure of a high-quality argumentative essay (e.g., Toulmin, 1958; Wingate, 2012). From the practical point of view, we had several meetings with teachers who were extensively involved in peer feedback activities for improving argumentative essay writing and they indicated that they expected to see that students clearly state their position in favor or against the topic and they are well aware of the responses that they can use to refute the possible counter-arguments (Table 2).

**Table 2:** The elements of the supported online peer feedback rubric for argumentative essay writing

Argumentative essay elements	Argumentative essay checker question prompt
Introduction on the topic	To what extent did your peer present a clear introduction on the topic in terms of motivation, importance, and the societal aspect of the issue at hand? What are your suggestions? Please explain.
Taking a position on the topic	To what extent did your peer present a clear position on the topic in favor or against the topic? What are your suggestions? Please explain.
Arguments for the position	To what extent did your peer provide arguments in favor of her/his own position on the topic? What are your suggestions? Please explain.
Justifications for arguments for the position	To what extent did your peer provide justifications (facts, evidence, examples, figures, experiences, etc.) for arguments in favor of her/his position? What are your suggestions? Please explain.
Arguments against the position (counter-arguments)	To what extent did your peer provide arguments against her/his position (counter-arguments) on the topic? What are your suggestions? Please explain.
Justifications for arguments against the position	To what extent did your peer provide justifications (facts, evidence, examples, figures, experiences, etc.) for arguments against her/his own position? What are your suggestions? Please explain.
Response to counter-arguments	To what extent did your peer respond (using justified arguments) to various counter-arguments against her/his position? What are your suggestions? Please explain.
Final conclusion and implications	To what extent did your peer come to a conclusion (restating her/his position) followed by a clear implication (suggestion and/or plan of action) for the position? What are your suggestions? Please explain.

After designing the peer feedback tool, in the next step, we focused on the implementation of the tool within a peer feedback and essay writing module. In this phase, it was decided to design and implement a module on the online learning platform called Brightspace. Brightspace is a cloud-based learning platform that is known as a user-friendly platform and the students were familiar with how to work with it. Therefore, students did not need any instructions on how to use Brightspace and how to follow the module and complete given tasks in the module. The online module was designed for three consecutive weeks to test the effectiveness and scalability of the suggested peer feedback tool. The module consisted of three main tasks and students performed one task each week. Before and during running the module, no training was given to students regarding how to give feedback. The reason behind this decision was to see if our suggested peer feedback tool can be effective, even though students do not have any training beforehand. This decision was in line with the goal of this study as we aimed to see to what extent this peer feedback tool can be applied on a large scale at different education levels and among different course domains.

In the first week, an introduction to the module was provided with instructions on how students should follow the module and what actions were expected from them (e.g., information on the research set-up of the study, expected goals and tasks for this module, instructions on how to follow the module, word limits for essays and feedback, deadlines, etc.). For their first task, students were asked to individually write an argumentative essay on one of the three controversial topics provided by the educators of each course. The topics were different for each course. For course A, the topics included, children and video games, Genetically Modified Organisms (GMOs), and Climate change. For example, on the topic of children and video games, students were asked to write an argumentative essay on whether they agreed with the idea that children should play video games or they should be banned from playing video games. Or in terms of GMOs, students were asked to write their opinion on whether humans should modify organisms’ genes or not. For course B, the topics were the use of RNAi-based biopesticide, the ban of glyphosates, and the use of gene drives for agricultural pest control. For course C, topics included the sugar tax, Covid-19 vaccines, and brain drain. For course D, topics were the long-term impacts of Covid-19 on the environment, the role of private actors in funding local and global biodiversity, and bans on the use of single-use plastics. Finally, for course E, the topics were as follows: scientists with links to the food industry should not be involved in risk assessment, powdered infant formula should be sterile, and preparation is the responsibility of the caregiver. All students in one course had an equal opportunity to select one topic among the three according to their preferences. The reason behind this was to reduce the risk of any potential bias with regard to students’ domain-specific knowledge on a specific topic because it was likely that some students could have extensive content knowledge on one specific topic, while others may not. Students were informed that their essay length should not exceed more than 800 words excluding the references. The first version of the essay written by the students was considered a pre-test.”

In the second week, students were invited to give written/asynchronous feedback and provide comments on two argumentative essays of their peers based on the designed supported peer feedback rubric (Table 2). This means that students were allowed to complete the peer feedback task at the time and pace of their choosing within a week. The reason behind this decision was to give time for students to better reflect on their peers’ essays as providing effective (peer) feedback is seen as a complex learning activity that causes a high cognitive workload (Valero Haro et al., 2019). The word number of comments for each element of argumentative essay writing was between 30 and 50 words. No specific recruitment strategy was used to pair students. That means that students were randomly assigned to dyads and they were asked to give feedback to each other. Students provided their feedback in the Brightspace platform using the FeedbackFruits tool. FeedbackFruits is an external EdTech tool embedded in Brightspace at Wageningen University and Research to drive students’ engagement through different peer collaboration strategies. This tool has many functionalities including peer review, assignment review, skill review, automated feedback, interactive video, interactive document, discussion assignments, interactive presentations, etc. By using this tool, teachers are able to create a rubric and ask students to reflect on their peers’ documents in different forms such as videos, pictures, reports, or essays. For this study, we used the peer review function which enables instructors to create assignments for students to provide asynchronous feedback to their peers. When students wrote their essays, in the second week, students were automatically assigned to complete the peer feedback task within a week at their own pace and time.

**Fig. 1:**
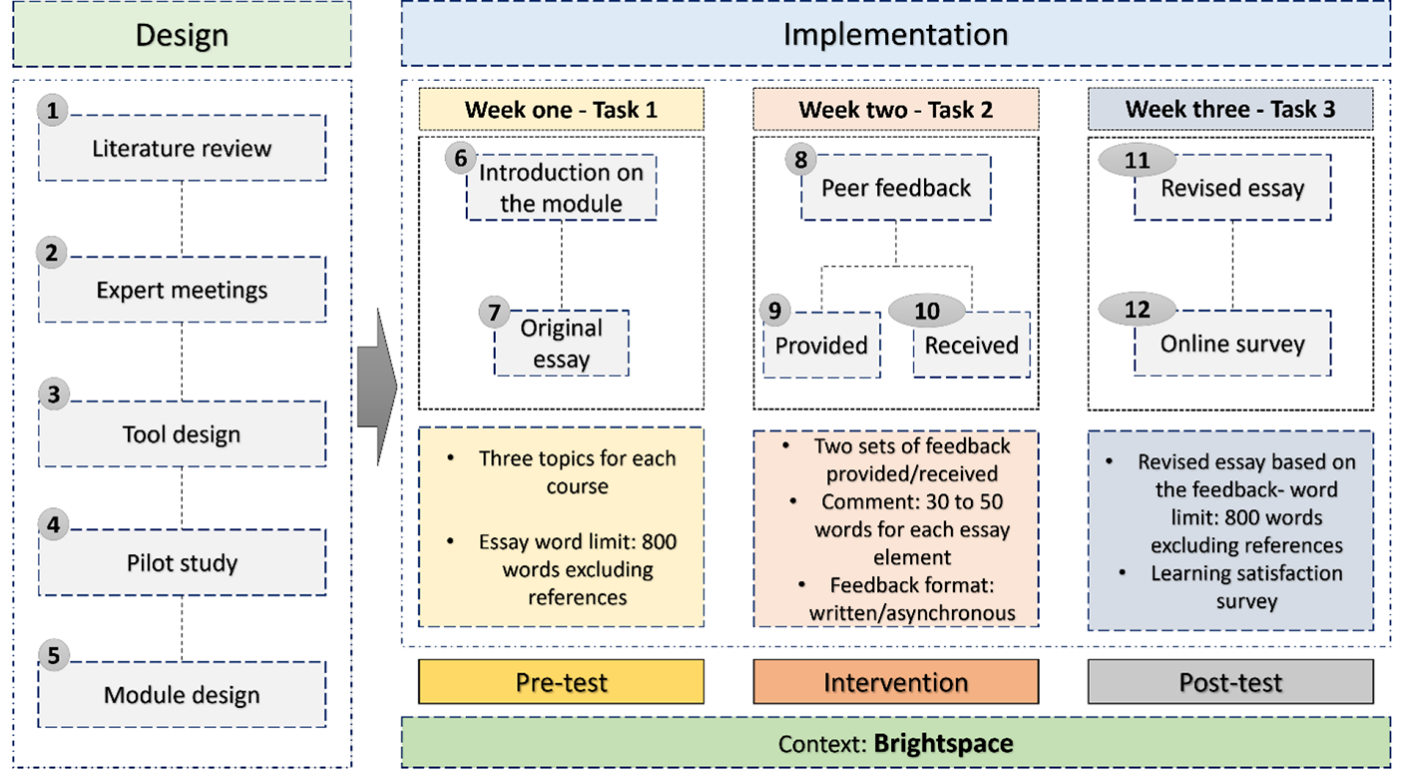
Design and implementation of the module

In the third week, students were requested to revise their first essay based on the received two sets of feedback from their two peers and submit their revised version of the essay in Brightspace. Similar to the original essay, students were informed that their revised essay length should not exceed more than 800 words excluding the references. The revised essay was considered as the post-test. After completing the module, students were asked to fill out an online survey about their learning satisfaction with the module (Fig. 1).

### Measurements

#### Argumentative essay writing performance

Students’ argumentative essay writing quality was assessed by an adjusted coding scheme built on the prior study of Noroozi et al. (2016). The coding scheme (Table 3) consisted of eight elements in line with the structure of high-quality argumentative essay Noroozi et al. (2016); Nussbaum & Edwards, 2011; Toulmin, 1958). The coding elements are (1) introduction on the topic, (2) taking a position on the topic, (3) arguments for the position, (4) justifications for arguments for the position, (5) arguments against the position (counter-arguments), (6) justifications for arguments against the position, (7) response to counter-arguments, and (8) conclusion and implications. These elements are scored from zero points (not mentioned) to three points (mentioned, elaborated, and justified). The mean score of all given points together determines students’ quality of argumentative essay writing performance. Five coders who were familiar with the coding scheme and argumentative essay writing worked together to analyze students’ essays in the pre-test and the post-test phases. The contingency coefficient analysis was used to determine the inter-rater reliability and the results showed that there is a reliable agreement between the coders (*p < 0.001*).

**Table 3:** Coding scheme to analyze the quality of students’ argumentative essay performance

Variables	Points	Labels for argumentative essay quality	Descriptions of the labels for argumentative essay quality
Introduction on the topic	Zero	Not mentioned at all	Introduction on the topic is not presented at all.
	One	Just mentioned	Introduction on the topic is just presented, but not elaborated and justified.
	Two	Mentioned and elaborated	Introduction on the topic is presented and elaborated, but not justified.
	Three	Mentioned, elaborated, and justified	Introduction on the topic is presented, elaborated, and justified.
Taking a position on the topic	Zero	Not mentioned at all	Position on the topic is not presented at all.
	One	Just mentioned	Position on the topic is just presented, but not elaborated and justified.
	Two	Mentioned and elaborated	Position on the topic is presented and elaborated, but not justified.
	Three	Mentioned, elaborated, and justified	Position on the topic is presented, elaborated, and justified.
Arguments for the position	Zero	Not mentioned at all	No argument in favour of the position is presented.
	One	Mentioned to a small extent	Only one argument in favour of the position is presented.
	Two	Mentioned to a moderate extent	Only two arguments in favour of the position are presented.
	Three	Mentioned to a great extent	More than two arguments in favour of the position are presented.
Justifications for arguments for the position	Zero	Not justified at all	Justification for arguments for the position is not presented at all.
	One	Justified to a small extent	Only one argument for the position is justified.
	Two	Justified to a moderate extent	Some but not all arguments for the position are justified.
	Three	Justified to a great extent	All arguments for the position are justified.
Arguments against the position (counter-arguments)	Zero	Not mentioned at all	No argument against the position is presented.
	One	Mentioned to a small extent	Only one argument against the position is presented.
	Two	Mentioned to a moderate extent	Only two arguments against the position are presented.
	Three	Mentioned to a great extent	More than two arguments against the position are presented.
Justifications for arguments against the position	Zero	Not justified at all	Justification for arguments against the position is not presented at all.
	One	Justified to a small extent	Only one argument against the position is justified.
	Two	Justified to a moderate extent	Some but not all arguments against the position are justified.
	Three	Justified to a great extent	All arguments against the position are justified.
Response to counter-arguments	Zero	Not mentioned at all	Response to counter-arguments is not presented at all.
	One	Just mentioned	Response to counter-arguments is just presented, but not elaborated and justified.
	Two	Mentioned and elaborated	Response to counter-arguments is presented and elaborated, but not justified.
	Three	Mentioned, elaborated, and justified	Response to counter-arguments is presented, elaborated, and justified.
Final conclusion and implications	Zero	Not mentioned at all	Conclusion and/or implications are not presented at all.
	One	Just mentioned	Conclusion and/or implications are just presented, but not elaborated and justified.
	Two	Mentioned and elaborated	Conclusion and/or implications are presented and elaborated, but not justified.
	Three	Mentioned, elaborated, and justified	Conclusion and/or implications are presented, elaborated, and justified.

#### Students’ learning satisfaction

To measure students’ learning satisfaction, an adjusted version of a questionnaire developed by Mahdizadeh (2008) was used. The adjusted version of the questionnaire consisted of four main categories and 24 items in total with a five-point Likert scale ranging from “almost never true = 1”, “rarely true = 2”, “occasionally true = 3”, “often true = 4”, to “almost always true = 5”. The first five items of this questionnaire assess students’ “perceived effects on the domain-specific learning outcomes”, and Items from 6 to 11 represent students’ “perceived effects on the domain-general learning outcomes”. Questions from 12 to 16 assess “ease of use of the module” and questions from 17 to 24 measure students’ “satisfaction with the learning task”. This questionnaire was used in some previous studies and its reliability for reuse is confirmed (e.g., Noroozi & Mulder, 2017). For this study, the validity of the survey was confirmed through a panel of experts including teachers, subject-matter experts, and educational research scholars.

### Analysis

The analysis of data was conducted in two phases. The first phase was qualitative analysis where we scored students’ qualitative data collected through argumentative essay writings in both the original and revised stages based on the coding scheme (see Table 3). This coding scheme was used to give a score to each element of the argumentative essay (e.g., score 2 to the introduction section which means: the introduction section is mentioned and elaborated) and also a total score for the whole essay. By such scoring, we were able to measure and analyze students’ progress from the original essay (pre-test) to the revised essay (post-test) and to see if this progress was significant or not.

In the second phase, quantitative analysis was adopted. In this phase, first descriptive statistics were performed to report mean and standard deviation for the variables (argumentative essay writing alone and with considering course domains and education levels from the pre-test phase to the post-test, and students’ learning satisfaction considering their course domains and education levels). Second, to control the effects of gender on the variables, we considered it as a covariate. To answer the first question we used the one-way MANCOVA for repeated measurement test to compare students’ progress in argumentative essay writing from pre-test to post-test. The two-way MANCOVA for repeated measurement test was used to address the second and third questions as we wanted to compare students’ progress in argumentative essay writing from the pre-test phase to the post-test in different course domains and education levels. To analyze the fourth research question, a one-way MANCOVA test was used for investigating students’ learning satisfaction with the online peer feedback module considering their course domains and education levels. Also, to compare every element of learning satisfaction in different courses, a pairwise comparison analysis was used to determine course differences in terms of learning satisfaction.

## Results

### RQ1

*To what extent does the supported online peer feedback module affect students’ argumentative essay writing performance?*

The results showed that the argumentative essay writing performance of all students has significantly improved from pre-test to post-test (Wilks’ λ = 0.65, F(7, 269) = 20.56, p < 0.01, Partial η2 = 0.35). This improvement was not only visible in the overall quality of the argumentative essay writing but also in all eight recognized elements of high-quality argumentative essay writing. Cohen (1988, pp. 280–287) suggests values of 0.01, 0.06, and 0.14 to indicate small, medium, or large effects for any measure of the proportion of variance explained. Accordingly, since the Partial η2 in argumentative essay writing performance from the pre-test to the post-test is higher than 0.14, it can be said that the effect size is large (Table 4).

**Table 4:** Students’ differences in mean scores of argumentative essay quality improvements from pre-test to post-test

Variables	Test	Mean	SD	Essay quality improvements of all students from pre-test to post-test
Introduction on the topic	Pre-test	2.74	0.52	F (1, 276) = 21.99, p < 0.01**, Partial η2 = 0.07
	Post-test	2.85	0.36	
Taking position on the topic	Pre-test	1.03	0.84	F (1, 276) = 135.6, p < 0.01**, Partial η2 = 0.33
	Post-test	1.57	0.89	
Arguments for the position	Pre-test	2.64	0.64	F (1, 275) = 5.57, p < 0.01**, Partial η2 = 0.02
	Post-test	2.80	0.6	
Justifications for arguments for the position	Pre-test	2.31	0.93	F (1, 276) = 31.76, p < 0.01**, Partial η2 = 0.10
	Post-test	2.55	0.75	
Arguments against the position (counter-arguments)	Pre-test	1.51	0.99	F (1, 276) = 77.05, p < 0.01**, Partial η2 = 0.21
	Post-test	1.84	0.88	
Justifications for arguments against the position	Pre-test	0.95	1.00	F (1, 276) = 86.35, p < 0.01**, Partial η2 = 0.23
	Post-test	1.42	0.98	
Response to counter-arguments	Pre-test	1.05	0.89	F (1, 276) = 47.02, p < 0.01**, Partial η2 = 0.14
	Post-test	1.31	0.87	
Final conclusion and implications	Pre-test	1.96	0.62	F (1, 276) = 52.93, p < 0.01**, Partial η2 = 0.16
	Post-test	2.24	0.51	
Overall argumentative essay writing	Pre-test	1.77	0.36	F (7, 269) = 20.54, p < 0.01**, Partial η2 = 0.35
	Post-test	2.06	0.34	

(P < 0.01)**, (P < 0.05)*

### RQ2

*To what extent does the supported online peer feedback module affect students’ augmentative essay writing performance with respect to their course domains?*

The results showed that there were no significant differences between students in their argumentative essay writing performance in different courses (Wilks’ λ = 0.88, F(28, 956.89) = 1.21, p = 0.20). This means that regardless of the course in which students participated, their argumentative essay writing has been improved from pre-test to post-test (Table 5).

**Table 5:** Course differences for argumentative essay writing performance improvements from pre-test to post-test

Variables	Test	Course										Course difference for essay quality improvements from pre-test to post-test
		Course A		Course B		Course C		Course D		Course E		
		Mean	SD	Mean	SD	Mean	SD	Mean	SD	Mean	SD	
Introduction on the topic	Pre-test	2.80	0.39	2.82	0.38	2.70	0.58	2.76	0.44	2.62	0.75	F (4, 272) = 0.75, p = 0.55
	Post-test	2.90	0.29	2.93	0.25	2.85	0.41	2.83	0.36	2.80	0.40	
Taking a position on the topic	Pre-test	0.94	0.72	0.75	0.91	1.04	0.78	1.11	0.81	1.14	1.01	F (4, 272) = 1.01, p = 0.40
	Post-test	1.51	0.72	1.03	1.01	1.67	0.96	1.65	0.82	1.68	0.95	
Arguments for the position	Pre-test	2.67	0.55	2.58	0.73	2.54	0.65	2.71	0.62	2.62	0.72	F (4, 271) = 2.15, p = 0.07
	Post-test	2.84	0.45	2.65	0.76	2.50	0.69	2.76	0.55	2.64	0.59	
Justifications for arguments for the position	Pre-test	2.32	0.87	2.13	1.05	2.23	0.94	2.47	0.87	2.22	0.95	F (4, 272) = 2.27, p = 0.06
	Post-test	2.78	0.57	2.51	0.78	2.34	0.84	2.66	0.67	2.32	0.86	
Arguments against the position	Pre-test	1.94	0.97	2.10	1.01	1.15	0.84	1.27	0.90	1.50	0.99	F (4, 272) = 0.73, p = 0.57
	Post-test	2.26	0.81	2.62	0.62	1.47	0.65	1.56	0.82	1.82	0.94	
Justifications for arguments against the position	Pre-test	1.11	1.16	1.72	1.16	0.78	0.86	0.84	0.86	0.72	0.92	F (4, 272) = 1.91, p = 0.10
	Post-test	1.82	1.07	2.34	0.76	1.10	0.82	1.23	0.81	1.14	1.01	
Response to counter-arguments	Pre-test	1.44	1.05	1.27	1.13	1.10	0.80	0.75	0.67	1.06	0.84	F (4, 272) = 0.23, p = 0.91
	Post-test	1.73	1.01	1.48	1.02	1.40	0.67	1.02	0.74	1.26	0.85	
Final conclusion and implications	Pre-test	2.13	0.34	2.00	0.70	1.86	0.68	1.92	0.64	1.94	0.71	F (4, 272) = 0.58, p = 0.67
	Post-test	2.32	0.47	2.20	0.41	2.23	0.56	2.21	0.55	2.26	0.48	
Overall argumentative essay writing	Pre-test	1.92	0.38	1.92	0.41	1.67	0.28	1.73	0.33	1.72	0.38	F (28, 956.89) = 1.21, p = 0.20
	Post-test	2.27	0.38	2.22	0.31	1.95	0.29	1.99	0.27	1.99	0.35	

(P < 0.01)**, (P < 0.05)*

### RQ3

*To what extent does the supported online peer feedback module affect students’ augmentative essay writing performance with respect to their education level (bachelor vs. master?*

The results showed that no significant differences between bachelor and master students were found in the mean score of essay quality improvements from pre-test to post-test (Wilks’ λ = 0.97, F(7, 268) = 1.24, *p* = 0.28). However, master students showed better improvements in justifications for arguments against the position (Table 6).

**Table 6:** Education level differences for argumentative essay writing performance improvements from pre-test to post-test

Variables	Test	Education levels				Difference between essay quality improvements of bachelor and master students from pre-test to post-test
		Bachelor		Master		
		Mean	SD	Mean	SD	
Introduction on the topic	Pre-test	2.75	0.49	2.74	0.56	F (1, 275) = 0.05, p = 0.47
	Post-test	2.84	0.37	2.87	0.33	
Taking a position on the topic	Pre-test	1.09	0.80	0.97	0.88	F (1, 275) = 0.99, p = 0.43
	Post-test	1.66	0.86	1.47	0.91	
Arguments for the position	Pre-test	2.66	0.63	2.63	0.65	F (1, 274) = 2.38, p = 0.12
	Post-test	2.68	0.60	2.72	0.59	
Justifications for arguments for the position	Pre-test	2.40	0.90	2.24	0.94	F (1, 275) = 0.98, p < 0.05*, Partial η2 = 0.02
	Post-test	2.56	0.74	2.54	0.76	
Arguments against the position	Pre-test	1.24	0.88	1.80	1.01	F (1, 275) = 0.73, p = 0.39
	Post-test	1.54	0.77	2.17	0.88	
Justifications for arguments against the position	Pre-test	0.82	0.86	1.09	1.13	F (1, 275) = 0.98, p < 0.05*, η2 = 0.02
	Post-test	1.19	0.81	1.67	1.09	
Response to counter-arguments	Pre-test	0.85	0.72	1.25	1.00	F (1, 275) = 0.24, p = 0.62
	Post-test	1.13	0.73	1.49	0.97	
Final conclusion and implications	Pre-test	1.91	0.65	2.03	0.59	F (1, 275) = 0.69, p = 0.40
	Post-test	2.22	0.55	2.27	0.46	
Overall argumentative essay writing	Pre-test	1.70	0.31	1.84	0.39	F (7, 268) = 1.24, p = 0.28
	Post-test	1.98	0.27	2.15	0.38	

(P < 0.01)**, (P < 0.05)*

### RQ4

*How do students in different course domains and education levels (bachelor vs. master) respond to the supported online peer feedback module in terms of their satisfaction?*

The results showed that there was a significant difference between bachelor and master students in terms of their learning satisfaction (Wilks’ λ = 0.93, F(4, 230) = 3.94, p < 0.01, Partial η2 = 0.06). Master students showed higher learning satisfaction than bachelor students. The higher satisfaction for master students was due to their perceived effects on the domain-general learning outcomes and satisfaction with the learning task. Since the Partial η2 in learning satisfaction between education levels is higher than 0.02, it can be said that the effect size is between small and medium (Table 7).

**Table 7:** Differences among bachelor and master students in terms of mean scores for learning satisfaction

Variables	Test	Mean	SD	Learning satisfaction differences among bachelor and master students
Perceived effects on the domain-specific learning outcomes	Bachelor	3.61	0.73	F (1, 233) = 2.70, p = 0.10
	Master	3.78	0.83	
	Total	3.70	0.79	
Perceived effects on the domain-general learning outcomes	Bachelor	3.44	0.72	F (1, 233) = 6.36, p < 0.05*, Partial η2 = 0.02
	Master	3.69	0.80	
	Total	3.57	0.77	
Ease of use of the module	Bachelor	4.05	0.76	F (1, 233) = 0.03, p = 0.84
	Master	4.03	0.78	
	Total	4.04	0.77	
Satisfaction with the learning task	Bachelor	3.49	0.61	F (1, 233) = 9.91, p < 0.01*, Partial η2 = 0.04
	Master	3.76	0.70	
	Total	3.63	0.67	
Overall Learning satisfaction	Bachelor	3.62	0.54	F (7, 269) = 20.54, p < 0.01**, Partial η2 = 0.06
	Master	3.80	0.63	
	Total	3.71	0.59	

(P < 0.01)**, (P < 0.05)*

Furthermore, the results showed that there were significant differences among students in different courses in terms of their learning satisfaction (Wilks’ λ = 0.73, F(16, 694.134) = 4.68, *p* < 0.01, Partial η2 = 0.07). These differences were found due to students’ different understanding of perceived effects on the domain-general learning outcomes and satisfaction with the learning task. Since the Partial η2 in learning satisfaction among students in different course domains is higher than 0.02, it can be said that the effect size is between small and medium (Table 8).

**Table 8:** Differences among students in different course domains in terms of mean scores for learning satisfaction

Variables	Test	Mean	SD	Pairwise Comparisons	Learning satisfaction differences among bachelor and master students
Perceived effects on the domain-specific learning outcomes	Course A	3.62	0.92	Course B > Course A*	F (4, 230) = 1.88, p = 0.11
	Course B	4.01	0.74	Course B > Course D*	
	Course C	3.68	0.78		
	Course D	3.58	0.70		
	Course E	3.81	0.76		
	Total	3.70	0.79		
Perceived effects on the domain-general learning outcomes	Course A	3.87	0.75	Course A > Course B**	F (4, 230) = 5.03, p < 0.01**, η2 = 0.08
	Course B	3.25	0.77	Course A > Course C*	
	Course C	3.46	0.73	Course A > Course D**	
	Course D	3.43	0.72	Course E > Course B**	
	Course E	3.80	0.77	Course E > Course C*	
	Total	3.57	0.77	Course E > Course D*	
Ease of use of the module	Course A	4.08	0.77	Course D > Course B*	F (4, 230) = 1.36, p = 0.24
	Course B	3.75	0.70	Course E > Course B*	
	Course C	3.97	0.89		
	Course D	4.08	0.70		
	Course E	4.15	0.82		
	Total	4.04	0.77		
Satisfaction with the learning task	Course A	3.80	0.74	Course A > Course C*	F (4, 230) = 2.86, p < 0.05*, η2 = 0.04
	Course B	3.64	0.52	Course A > Course D*	
	Course C	3.45	0.70	Course E > Course C*	
	Course D	3.51	0.57	Course E > Course D*	
	Course E	3.80	0.75		
	Total	3.63	0.67		
Overall Learning satisfaction	Course A	3.84	0.65	Course A > Course D*	F (16, 694.134) = 4.68, p < 0.01**, η2 = 0.07
	Course B	3.64	0.53	Course E > Course C*	
	Course C	3.61	0.63	Course E > Course D*	
	Course D	3.63	0.50		
	Course E	3.88	0.66		
	Total	3.71	0.59		

(P < 0.01)**, (P < 0.05)*

## Discussions

This study was conducted to examine the effectiveness of the designed supported peer feedback module to improve students’ argumentative essay writing performance in online learning environments on a large scale in a higher education context. To be able to scale up the findings of this study to a large number of students with different domain knowledge backgrounds at bachelor and master levels, we tested our module on a group of students from five different course domains at bachelor and master levels. We also investigated students’ satisfaction with the learning experiences in the module.

### Discussion on RQ1

Our findings for the first research question revealed that there was an increase in the quality of writing argumentative essays in all elements from the original essay (pre-test) to the revised essay (post-test). That means that by use of the suggested peer feedback tool, students gave effective feedback on the original argumentative essay of their peers, and implementation of such feedback improved students’ essay quality in the revised version. This improvement was significant for all elements of the argumentative essay structure (e.g., introduction on the topic, taking a position, and arguments). These findings are in line with the prior studies where positive impacts of peer feedback strategies on promoting students’ argumentative essay writing performance were reported (e.g., Noroozi et al., 2022; Kerman et al., 2022; Latifi et al., 2020, 2021; Ramon-Casas et al., 2019). For example, Ramon-Casas et al. (2019) reported that their peer-feedback strategy made a significantly positive difference between students’ first and second essays. In another study, Noroozi et al. (2016) reported similar findings where their adopted peer feedback module has helped students to enhance argumentative essay writing skills. The main reason to explain this finding can be related to the quality of the provided peer feedback tool for students as it was built on evidence from both theory and practice. It should be also noted that although our findings are in line with and supported by the prior studies (e.g., Latift et al., 2021), what makes the present study stand out compared to the previous studies is that the current findings support the potential of the peer feedback tool in this study for students from different course domains at both bachelor and master levels.

### Discussion on RQ2

The findings related to the second research question showed no significant differences in the improvement of argumentative essay writing performance between students with different course domain knowledge levels. This finding indicates that the designed online supported peer feedback module significantly improved students’ argumentative essay writing regardless of their course domain knowledge. This finding supports the scalability of the suggested supported online peer feedback module in this study for enhancing students’ argumentative essay writing skills in different courses regardless of the course domain. Several prior studies reported influential impacts of students’ course domain knowledge on their peer feedback and argumentation performance (Alqassab et al., 2018; Patchan et al., 2016; Van Zundert et al., 2012). For example, Algassab et al. (2018) reported that students with high-level domain knowledge provide more self-regulative feedback while students with low-level domain knowledge delivered peer feedback at a task level. Or Valero Haro et al. (2022) found that the quality of students’ domain-specific knowledge is positively correlated with their successful argumentation performance. One of the reasons that our findings conflict with prior studies on this aspect is that peer feedback tools used in the mentioned prior studies were course-domain specific (Alqassab et al., 2018). This means that the success of prior peer feedback tools was dependent on students’ domain-specific knowledge. While, in contrast, our peer feedback tool is designed based on the structure of high-quality argumentative essay writing and this structure does not differ from one course to another (Toulmin, 1958). Therefore, our results support the claim that aspects of argumentation can be transferred from one course to another and if students receive support and guidance for the structure of the arguments, their argumentation competence can be improved (Noroozi et al., 2018). An additional reason for this finding is that peer feedback is naturally a process-oriented pedagogical activity (Kerman et al., 2022; Shute, 2008), which means that peer feedback activities, regardless of their types, encourage students to be involved in critical and active collaborative learning with peers on a specific topic where higher-order activities such as criticizing, reflecting, analyzing, and evaluating are often used (Liu & Carless, 2006; Topping, 2009). We speculate that students’ deep involvement in such knowledge-shared and active learning processes could be an additional reason why students’ argumentation performance was improved in all course domains.

### Discussion on RQ3

It was also found that the designed online supported peer feedback module has helped both bachelor and master students in improving their argumentative essay quality from the first essay to the second essay. This conveys that despite the differences we have pointed out between bachelor and master students such as differences in learning experiences and strategies, motivation, academic writing purposes, and personal beliefs (Van Seters et al., 2012; Yu et al., 2019), the supported peer feedback module was found effective in enhancing both bachelor and master students’ argumentation skills in essay writing. Only a difference was found in providing justifications for arguments against the position in which master students performed better than bachelor students. A reason for this outperformance might be that master students have more experience with academic writing (Yu et al., 2019). In general, the findings indicate that our suggested peer feedback module could be used for students of different degrees. This finding is supported by a few prior studies such as Aghaee and Keller (2016) where the authors reported positive impacts of ICT-supported peer interaction on improving both bachelor and master thesis processes. In line with the findings on course domains, it seemed that the improvement in the performance of both bachelor and master students is due to the concertation of our peer feedback module on the structure of high-quality argumentative essays, rather than on the content of the essays, which make it easier for students to apply their argumentation competences (Noroozi et al., 2018).

### Discussion on RQ4

Finally, we found that master students were more satisfied with the module compared to bachelor students. The results showed that master students perceived the learning module as more effective for their domain-general learning outcomes. Master students’ higher satisfaction with domain-general learning outcomes could be due to their better realization of the importance of argumentation in their academic efforts as they have grown their academic attitude. Master students usually have more learning experiences than bachelor students and they probably know that argumentation knowledge is critical for academic success (Van Seters et al., 2012; Yu et al., 2019). Therefore, they might have better realized the importance of such a module and the benefits that they can get from improving their argumentation skills compared to bachelor students.

In addition, differences were found in students’ learning satisfaction among different course domains. Similarly, these differences were mainly due to students’ perceived effects on the domain-general learning outcomes. Comparable with our findings for learning satisfaction for bachelor and master students, here, it can also be seen that the highest learning satisfaction belongs to course A (M = 3.87, SD = 0.75) and course E (M = 3.80, SD = 0.77). Both these courses are delivered at the master level. Therefore, the reason we provided for the bachelor and master students’ differences in learning satisfaction can be also applied here. This means that the differences in learning satisfaction of students in different course domains could be related to their realization of the importance of the module and the advantages of this module in their academic and professional growth. This finding is in line with our prior report on the effectiveness of the suggested peer feedback module in improving students’ domain-general knowledge as it focuses on the structure of the argumentation rather than the content of the essay (Noroozi et al., 2018).

## Limitations and implications for future research and practice

There are some limitations to this study that should be acknowledged. In this study, we only considered students’ domain knowledge and education levels as the variables that can influence students’ performance in argumentative essay writing. However, students’ other characteristics such as their epistemic beliefs (Noroozi, 2022), and culture (Tsemach & Zohar, 2021) can also influence their argumentative essay writing performance. Future studies should investigate the intersection impacts of epistemic beliefs and culture in peer feedback and argumentative essay writing performance. In this study, students in each course had a choice to select one topic among the three offered topics. It is possible that the selection of a topic based on students’ choices may have influenced the findings of this study. Therefore, the findings of this study should be interpreted with respect to this matter. For future studies, we suggest exploring how different topics may result in different received feedback patterns and uptake among successful, less successful, and unsuccessful students. In addition, while our study expands our understanding of peer feedback impacts on students’ argumentation competence in essay writing, we did not dig into the impacts of the feedback features (Patchan et al., 2016; Wu & Schunn, 2020) on students’ argumentative essay writing performance. For future studies, we suggest exploring the relationships between the features of peer feedback such as affective, cognitive, and constructive features (e.g., Patchan et al., 2016; Wu & Schunn, 2020) and students’ argumentative essay writing performance. In addition, we did not find any empirical studies to support the findings of this study at a large scale. However, as mentioned earlier there is some theoretical evidence to support the findings of this study (see Noroozi et al., 2018). By saying this, we suggest testing our supported peer feedback module again in different course domains at the bachelor and master levels to investigate the reliability of the module to be scaled up. Finally, the higher learning satisfaction of master students indicates that maybe the module needs to be redesigned in a way to be more in line with bachelor students’ learning expectations. It is suggested to explore this in future studies.

Although we acknowledged the limitations of this study, the findings of this study are valuable for future educational practice in the context of argumentative essay writing within online higher education settings. The most important takeaway message of this study for teachers is that argumentation structure stands independently from students’ course domain knowledge and their education level and that it can be learned and transferred to different contexts. Therefore, teachers can use the suggested peer feedback tool in this study to improve students’ argumentation performance with respect to its structure in any course and education level. Such use could add two educational values. First, this helps with decreasing teachers’ workload in terms of providing feedback. Because, instead of giving one-by-one teacher feedback, students take responsibility to give peer feedback based on the use of this tool. Second, by using this tool, students are automatically involved in an active and collaborative peer learning process where they learn about other peers’ work, critically review it, and learn from it.

## Conclusion

This study was a response to a research gap in the literature regarding the scalability of existing peer feedback tools for argumentative essay writing as they were in general content-oriented and not applicable to other contexts. Our research was built on the idea that argumentation knowledge can be transferred across different disciplines and educational levels (Noroozi et al., 2018). In this study, we designed and developed a peer feedback tool that was based on the structure of argumentative essay writing and we tested our suggested tool in different course domains and education levels. The findings of this study confirm the effectiveness of the tool in improving all students’ argumentation performance in essay writing. These findings are indications of the effectiveness of the tool at a large scale and add value to the existing literature regarding the scalability of the peer feedback tools for argumentative essay writing in online higher education. The findings are important for future online education practice as our study proposes a peer feedback tool for teachers that can be used in different contexts for complex skills, particularly argumentative essay writing.

## Data Availability

The datasets generated during and/or analyzed during the current study are available from the corresponding author upon reasonable request.
